# A recombinant oncolytic Newcastle virus expressing MIP-3α promotes systemic antitumor immunity

**DOI:** 10.1136/jitc-2019-000330

**Published:** 2020-08-05

**Authors:** Feng-Ying Huang, Jin-Yan Wang, Shu-Zhen Dai, Ying-Ying Lin, Yan Sun, Liming Zhang, Zhuoxuan Lu, Rong Cao, Guang-Hong Tan

**Affiliations:** Key Laboratory of Tropical Translational Medicine of Ministry of Education & Hainan Provincial Key Laboratory of Tropical Medicine, Hainan Medical University, Haikou, Hainan, China

**Keywords:** immunology, oncology

## Abstract

**Background:**

The oncolytic Newcastle disease virus (NDV) is inherently able to trigger the lysis of tumor cells and induce the immunogenic cell death (ICD) of tumor cells and is also an excellent gene-engineering vector. The macrophage inflammatory protein-3α (MIP-3α) is a specific chemokine for dendritic cells (DCs). Thus, we constructed a recombinant NDV expressing MIP-3α (NDV-MIP3α) as an in vivo DC vaccine for amplifying antitumor immunities.

**Methods:**

The recombinant NDV-MIP3α was constructed by the insertion of MIP-3α cDNA between the P and M genes. Western blotting assay and ELISA were used to detect MIP-3α, HMGB1, IgG, and ATP in the supernatant and sera. The chemotaxis of DCs was examined by Transwell chambers. The phenotypes of the immune cells (eg, DCs) were analyzed by flow cytometry. The antitumor efficiency of NDV-MIP3α was observed in B16 and CT26 tumor-bearing mice. Immunofluorescence and immunohistochemistry were applied to observe the ecto-calreticulin (CRT) and intratumoral attraction of DCs. Adoptive transfer of splenocytes and antibodies and depletion of T-cell subsets were used to evaluate the relationship between antitumor immunities and the role of the T-cell subtype.

**Results:**

The findings show that NDV-MIP3α has almost the same capabilities of tumor lysis and induction of ICD as the wild-type NDV (NDV-WT). MIP-3α secreted by NDV-MIP3α could successfully attract DCs in vitro and in vivo. Both B16 and CT26 cells infected with NDV-MIP3α could strongly promote DC maturation and activation. Compared with NDV-WT, intratumoral injection of NDV-MIP3α and the adoptive transfer of T lymphocytes from mice injected with NDV-MIP3α resulted in a significant suppression of B16 and CT26 tumor growth. The NDV-MIP3α-induced production of tumor-specific cellular and humoral immune responses was dependent on CD8^+^ T cells and partially on CD4^+^ T cells. A significant reversion of tumor microenvironments was found in the mice injected with NDV-MIP3α.

**Conclusions:**

Compared with NDV-WT, the recombinant NDV-MIP3α as an in vivo DC vaccine demonstrates enhanced antitumor activities through the induction of stronger system immunities and modulation of the tumor microenvironment. This strategy may be a potential approach for the generation of an in vivo DC vaccine.

## Introduction

1. Oncolytic viruses (OVs) can infect and replicate in tumor tissues, leading to direct lysis of tumor cells. Recently, talimogene laherparepvec, a modified herpes virus encoding the granulocyte-macrophage colony-stimulating factor (GM-CSF), was approved for the treatment of advanced melanoma.^1–3^ Besides the direct lysis of tumor cells, many new mechanisms related to OV-mediated therapy of cancers have been reported. Notably, oncolytic virotherapy combined with immune-checkpoint inhibitor enhances the clinical benefit by reversing the suppressive tumor microenvironment, which has been evidenced in several preclinical and clinical trials.^4–7^ In addition, increasing evidence shows that OVs can induce the immunogenic cell death (ICD) of cancer and stromal cells through the release of damage-associated molecular patterns (DAMPs) and tumor-associated antigens.^8–11^ As model pathogens, OVs can improve the effects of ICD by simultaneously promoting the stimulation of pathogen-associated molecular patterns (PAMPs) and the production of inflammatory cytokines.^12–14^ Currently, the hallmark DAMP molecules include the surface-exposed calreticulin (ecto-CRT), ATP, and the high mobility group box 1 (HMGB1).^15^ Dendritic cells (DCs) constitutively express DAMP receptors on their surface, including CD91 for CRT, TLR4 for HMGB1, and P2RX7 for ATP.^16–18^ After secretion, the DAMP molecules bind to their corresponding receptors on DCs, leading to an improved antigen uptake and capacity to stimulate the T cells.

The immune-suppressive microenvironment is one of the major barriers for priming effective antitumor immunity.^19–21^ To escape immune surveillance and evasion, tumor cells can express suppressive molecules (eg, programmed death-ligand 1 (PD-L1) and Fas Ligand (FasL)) on their surface, release inhibitory cytokines (eg, TGF-β and IL-10), inhibit tumor-specific T-cell expansion, or even directly promote T-cell apoptosis and proliferation.^22–24^ In addition, tumor cells can recruit and expand various types of inhibitory tumor-infiltrating lymphocytes (TILs), including tolerogenic DCs, regulatory T cells (Tregs), myeloid suppressor cells, and tumor-derived macrophages.^25–27^ Thus, a new tumor vaccine to remodulate the suppressive microenvironments as far as possible back to normal microenvironments may be promising for tumor immunotherapy in the future.

DCs are the most powerful antigen-presenting cells. A general approach for the generation of DC-based vaccines is to pulse tumor lysates or tumor-related antigens with DCs ex vivo and inject these treated DCs back into the patients’ tumors.^28^ The ex vivo procedures for the generation of DC vaccines are complex and difficult to standardize. Thus, we have previously considered an in vivo strategy by which endogenous DCs are attracted to the tumor sites using a live tumor cell to express a DC chemokine, the macrophage inflammatory protein-3α (MIP-3α or CCL20), which proved to be effective in several murine tumor models.^29^

Targeted lysis of tumor cells by OVs results in ICD of tumor cells, the release of tumor antigens, and the secretion of type I interferon (IFN-α), and all of these factors can efficiently promote DC maturation and cross-presentation of tumor antigens.^30 31^ In addition, OVs can be used as gene-engineering vectors to express active molecules.^32^ The oncolytic Newcastle disease virus (NDV) is reported to have all of these characteristics.^31–33^ Thus, in this study, NDV was used as a carrier to express MIP-3α (hereafter named NDV-MIP3α). After NDV-MIP3α was shown to express and secrete active MIP-3α in vivo effectively, we established two tumor models to investigate whether NDV-MIP3α could better induce tumor lysis and promote systemic antitumor immunity. The results demonstrate that NDV-MIP3α is clearly superior to the wild-type NDV (NDV-WT) in the induction of antitumor immunity.

## Methods

### Generation of the recombinant NDV-MIP3α and detection of virus replication

The lentogenic NDV LaSota strain was used to generate the recombinant NDV-MIP3α and wild-type (NDV-WT) control for all of the experiments, as previously reported.^32^ Briefly, the DNA fragment encoding murine MIP-3α was isolated from the plasmid pORF5-MIP-3α (InvivoGen, San Diego, California, USA), and then flanked with the appropriate NDV-specific RNA transcriptional signals. After that, the flanked MIP-3α DNA was inserted into the SacII site between the P and M genes of pT7NDV/LS. The recombinant NDVs were rescued and sequenced with reverse transcription-PCR to confirm the insertion integrity. The rescued NDVs were passaged in embryonated hen eggs. Virus titers were measured by immunofluorescence in A549 cells after serial dilution. To detect the virus replication kinetics, 5×10^4^ B16 cells were infected with input virus (1×10^3^ pfu) in 12-well plates at a 1 multiplicity of infection (MOI) in 100 µL. After 1 hour incubation, the media was replaced with 2 mL of fresh media, and the cells were continually incubated at 37°C in Dulbecco's modified eagle medium (DMEM, 1 mL) with tosyl-phenylalanine chloromethyl-ketone (TPCK) trypsin (250 ng/mL). The culture supernatants were collected at 24, 48, 72, and 96 hours to examine the virus replication by immunofluorescence in A549 cells, as previously reported.^32^

### Cytotoxicity assays

Cytotoxicity assays were performed to evaluate the ability of the OV or splenic lymphocytes to kill tumor cells using the CytoTox 96 kit (Promega, Madison, Wisconsin, USA), as previously described.^29 32^ For the lysis of B16 cells, infections were cultured and treated as aforementioned for the detection of the virus titers, and B16 cells were infected in 6-well plates at 25% confluency. After infection, cells were collected at 24, 48, 72, and 96 hours, and further incubated with Triton X-100 (1%) at 37°C for 30 min. The lactate dehydrogenase activity in the lysates was determined.^32^ For the detection of cytotoxic T lymphocytes (CTLs), tumor cells (target) in logarithmic growth were first plated on 96-well plates. Then the splenic lymphocytes (effector) were added to the plates to a total volume of 100 µL in a series of E (effector):T (target) ratios (5–40:1). The plates were further cultured in a 5% CO_2_ atmosphere at 37°C for 8 hours. Fifty-microliter aliquots were transferred to another 96-well plate, and then the reconstituted substrate was added to a final volume of 100 µL. After incubation for 60 min in the dark at room temperature, stop solution (50 µL) was added to each well, and the values of optical density were detected at 492 nm by an ELX808IU microplate system (Bio-Tek, Winooski, Vermont, USA). The percentage of target cell death at each E:T ratio was calculated, as reported previously.^29^

### Western blotting

MIP-3α, HMGB1, and sera IgG were analyzed by western blotting. Tumor cells were infected with NDV-MIP3α or NDV-WT at the indicated MOI for 24 hours, and the cultured supernatants or cell lysates were collected. In addition, the sera from the tumor model mice were collected. The supernatants, cell lysates, or sera were separated in 10% sodium dodecyl sulfate polyacrylamide gel electrophoresis (SDS-PAGE) gels. The proteins were then transferred onto a polyvinylidene difluoride (PVDF) membrane (Bio-Rad) using a Mini Transblot system (Bio-Rad, Hercules, California, USA). Thereafter, the PVDF membranes were blocked at 4°C in 10% non-fat milk, washed three times with PBS, and then probed with antibodies (Abcam) targeting murine MIP-3α, HMGB1, or IgG at a 1:150 dilution. The membranes were incubated in enhanced chemiluminescence (ECL) substrates A and B (1:1) for 30 min, and the resultant images were captured by an ECL system (Amersham Biosciences, UK). The band densities were semiquantitatively analyzed by ImageJ V.1.8.0.

### In vitro DC chemotaxis assay

The in vitro DC chemotaxis assay was carried out in pore polycarbonate filters of 24-well Transwell chambers (Corning Costar, New York, USA), as previously reported.^29^ Briefly, the top chamber was covered with 5×10^4^ DCs in 200 µL of BSA medium (0.5%), and the lower chamber was filled with the indicated percentages of supernatants from NDV-MIP3α-treated, NDV-WT-treated, or PBS-treated tumor cells to a volume of 750 µL. The Transwell chambers were incubated at 37°C for 3 hours in a 5% CO_2_ atmosphere. Thereafter, the filter between the top and lower chamber was washed by Hank's balanced salt solution (HBSS), fixed, and stained on a slide. The number of chemotactic DCs on the lower chamber was recorded by a microscope system (MT20, OSIS, Germany) at 200× magnification. The results of five high-power fields were quantified.

### Flow cytometric analysis

Flow cytometry (FCM) was performed as previously reported.^29^ To isolate the spleen lymphocytes, spleens were pressed through 100 µm cell strainers (BD Biosciences, Franklin Lakes, New Jersey, USA), and the erythrocytes were lysed with ACK buffer. The cell suspensions were then filtered through a cell strainer (100 µm). Tumor tissues were minced with scissors and digested in culture medium with 0.8 mg/mL Dispase II, 0.2 mg/mL collagenase P, 0.1 mg/mL DNase I (all from BD Biosciences, San Jose, California, USA) for 30 min at 37°C. Isolated cells from B16 and CT26 tumors were filtered through a cell strainer (70 µm) and purified on Ficoll gradient. Splenocytes or lymphocytes from tumor tissues (1×10^6^) were stained with the corresponding monoclonal antibodies conjugated with fluorescein isothiocyanate (FITC), phycoerythrin (PE), allophycocyanin (APC), or Percp-Cy5.5 for 30 min at 4°C. The monoclonal antibodies included anti-CD11c, anti-DEC205, anti-CD80, anti-CD86, anti-IFN-γ, anti-TNF-α, anti-CD4, anti-CD8, anti-CD25, and anti-FOXP3, which were purchased from BD Biosciences (Franklin Lakes, New Jersey, USA) or eBioscience (San Diego, California, USA). For intracellular cytokine staining, 2×10^6^ splenocytes or lymphocytes from tumor tissues were stimulated with tumor lysates (5 µg/mL) in culture medium with 10% FCS and 2 µg/mL brefeldin A (BD Bioscience) for 6 hours at 37°C. Intracellular cytokine staining was performed using the Cytofix/Cytoperm kit (BD Bioscience) according to the manufacturer’s protocol. The stained cells were detected by FCM (FACS Canto II, BD Biosciences), and the data were analyzed with FlowJo software V.10 (Tree Star, Ashland, Oregon, USA).

### ELISA and enzyme-linked immunospot (ELISPOT) assay

The HMGB1 and ATP in the supernatants from the virus-infected or PBS-treated tumor cells and the IgG antibody in the pooled sera from the tumor-bearing mice were detected by commercially available ELISA kits (Wuhan Boster Biological Technology, China), according to the manufacturer’s protocol as described previously.^29^ In addition, ELISPOT was performed to detect the numbers of splenic mononuclear cells that secreted the IgG antibodies specific against tumor cells, as previously reported.^29^ Briefly, 30 mg/mL of tumor lysate was coated to the PVDF-bottomed 96-well Filtration Plates (Millipore, Billerica, Massachusetts, USA). After that, the splenic mononuclear cells isolated from the spleen tissues of the mice injected with NDV-MIP3α, NDV-WT, or PBS were cultured on the plates at 37°C in an atmosphere of 5% CO_2_ for 6 hours. The number of IgG-secreted cells was counted as the number of spots that were stained with alkaline phosphatase-labeled anti-mouse IgG antibodies (Wuhan Boster Biological Technology).

### Immunofluorescence

Immunofluorescence was performed to detect the presence of ecto-CRT on the cell surface, as previously reported.^34^ Briefly, tumor cells (1×10^5^/well) were incubated into 6-well plates and infected with 1 MOI NDV-MIP3α, 1 MOI NDV-WT, or treated with only PBS. After a 10 min incubation at 37°C in a 5% CO_2_ atmosphere, the cells were washed three times with PBS and fixed in 4% paraformaldehyde at room temperature for 30 min. The fixed tumor cells were labeled with an anti-CRT antibody (1:500) at 4°C overnight and then washed three times with cold phosphate buffer saline (PBS). After that, the cells were stained with a FITC–labeled secondary antibody (1:500) for 1 hour at room temperature. At the same time, the cell membrane and nuclei were stained with DiI (MedChemExpress, USA) and 4',6-diamidino-2-phenylindole (DAPI, Beyotime Biotechnology, China) for 10 min, respectively. Pictures were obtained with a confocal microscope (FV1000, Olympus, Japan).

### Tumor models

The mice for tumor models in this study were authorized by the Animal Use and Care Committee of Hainan Medical University. Mice at 6–8 weeks of age were used to establish tumor models in which 5×10^5^ tumor cells were intradermally implanted in the right flank. To observe the antitumor efficacy of the NDV-MIP3α injection, tumor-bearing mice were divided into three groups (10 mice in each group) and intratumorally injected with NDV-MIP3α (2×10^7^ pfu in 100 µL), NDV-WT (2×10^7^ pfu in 100 µL), or PBS (100 µL) on day 7 after tumor cell injection, as previously described.^32^ The B16 and CT26 tumor models were established in C57BL/6 and BALB/c syngeneic mice, respectively. The tumor images and volumes were directly captured by a handheld device (TM900, Peira Scientific Instruments, Belgium) in a 3-day interval.

### Immunohistochemistry

Immunohistochemistry was performed to detect CD11c and DEC205 double-positive DCs in tumor tissues, as previously reported.^35^ Briefly, frozen sections (5 µm) from tumor tissues 3 days after the injection of NDV-MIP3α, NDV-WT, or PBS were placed on the slides, fixed in acetone for 15 min, and air-dried for 50 min. After washing in PBS three times, the slides were incubated with PBS combined with 5% normal goat serum for 60 min at room temperature, then incubated overnight at 4°C with rat anti-mouse antibodies against CD11c and DEC205 (eBioscience) at a 1:50 dilution. After that, the sections were further stained with streptavidin biotin-labeled reagents (Dako LSAB kit, peroxidase; Dako), and the slide images were captured under a microscope (MT20, OSIS, Germany).

### Adoptive transfer of splenocytes and antibodies

Adoptive transfers were performed as previously reported.^29^ Briefly, the spleens of the tumor-bearing mice were collected 14 days after the injection of NDV-MIP3α (2×10^7^ pfu in 100 µL), NDV-WT (2×10^7^ pfu in 100 µL), or PBS (100 µL). The recipient mice (n=5) were injected with 3×10^6^ splenocytes per mouse through the tail vein, and the mice were inoculated with B16 cells (5×10^5^ per mouse) in the right flank on the same day. After that, the transfers were repeated two more times in 5-day intervals: once on day 5 and again on day 10 after tumor cell inoculation. On day 18, after tumor cells were injected, the tumor images and volumes were directly captured by a TM900 system (Peira Scientific Instruments, Belgium). To adoptively transfer antibodies, we first collected the sera from tumor-bearing mice intratumorally injected with NDV-MIP3α, NDV-WT, or PBS on days 14–25. The purified immunoglobulins were acquired after the pooled sera were passed through a chromatography column (CM Affi-gel Blue Gel Kit; Bio-Rad). After that, the antitumor effects on the adoptive transfer of the immunoglobulins in vivo were evaluated. C57BL/6 mice were intradermally implanted with 5×10^5^ B16 cells in the right flank, and each mouse was then injected through the tail vein with the purified immunoglobulin (50 mg/kg) on the same day. Two additional transfers of immunoglobulins were carried out on days 5 and 10 after tumor cell inoculation. On day 18, after tumor cells were inoculated, the tumor images and volumes were also captured by the TM900 system.

### Depletion of CD4^+^ or CD8^+^ lymphocytes

Cell depletions were performed as previously reported.^29^ In short, a monoclonal rat anti-CD4 IgG (clone GK 1.5) or an anti-CD8 IgG (clone 2.43) was isolated and subsequently purified from the corresponding expressing hybridoma cells (ATCC, Manassas, Virginia, USA). Each mouse was first injected intraperitoneally with 1 mg of monoclonal antibodies on day 0, and the dose was repeated four more times (five in total) in 4-day intervals. The spleen cells were analyzed by FCM (FACS Canto II, BD Biosciences), and the efficacy of cell depletion was greater than 98%. Thereafter, the depleted mice were inoculated with B16 tumor cells (5×10^5^) on day 3 (after the antibody injection) and intratumorally injected with NDV-MIP3α, NDV-WT, or PBS on day 6. The tumor images and volumes were captured by a TM900 system, as described above. The titers of IgG antibodies specific to B16 tumor cells in the sera were detected by ELISA on day 21 (18 days after tumor cell inoculation), as aforementioned.

### Statistical analysis

The data in this study were analyzed by GraphPad Prism software (V.6). A non-parametric two-tailed Student’s t-test and one-way or two-way analysis of variance followed by a Bonferroni’s post hoc test were performed to analyze the difference of data from two samples and compare more than two samples, respectively. Data are shown as mean±SD. P<0.05 indicates a statistically significant difference.

## Results

### Generation and evaluation of the recombinant virus NDV-MIP3α

**Figure 1:**
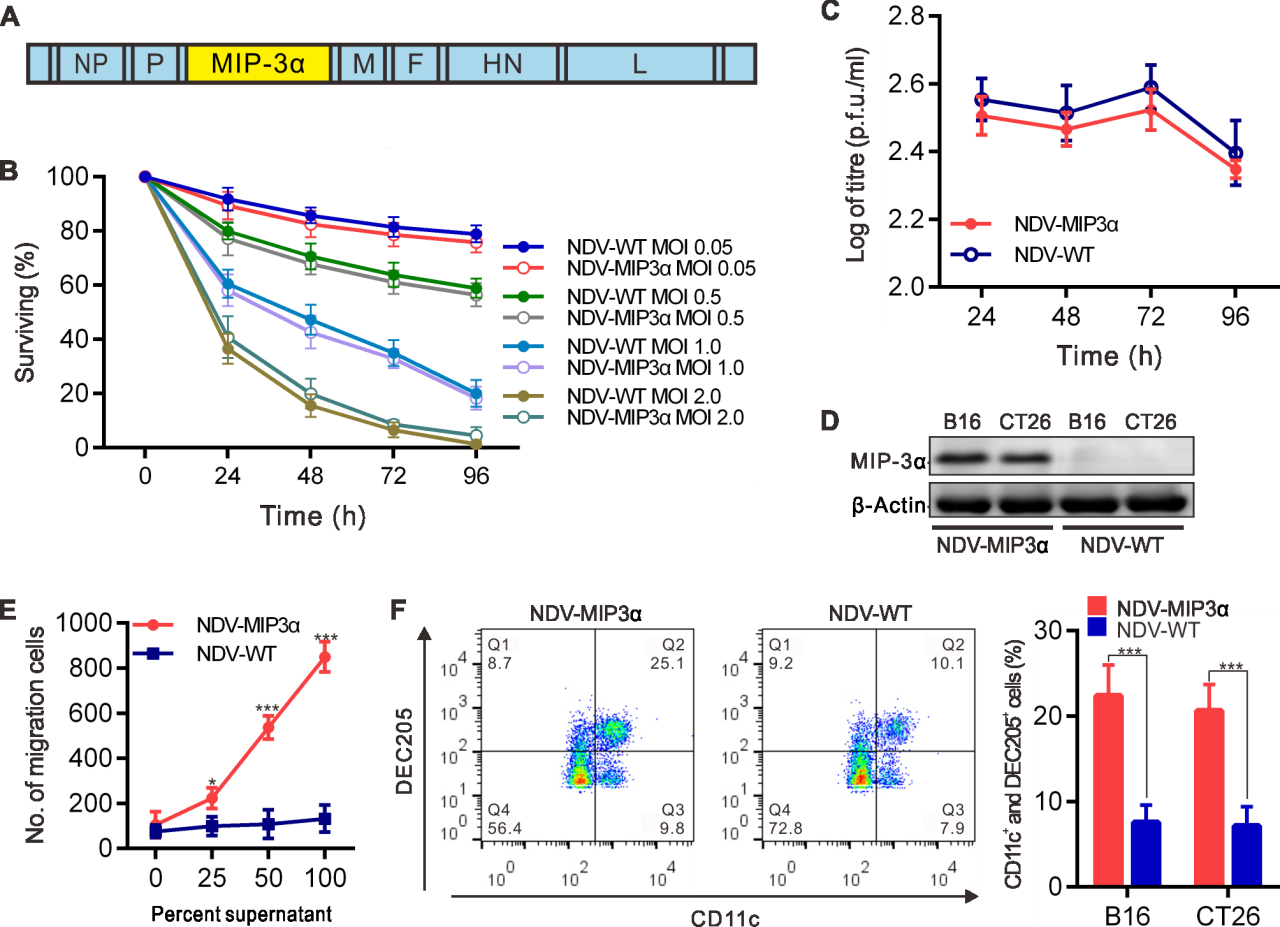
Generation and evaluation of the recombinant virus NDV-MIP3α. (A) Schematic presentation of the recombinant NDV-MIP3α, showing the insertion site of the MIP-3α cDNA. (B) Lysis of B16 cells by NDV-MIP3α and NDV-WT at the indicated MOI and time points. (C) Virus replication of 1 MOI NDV-MIP3α or NDV-WT at the indicated time points in B16 cells. (D) MIP-3α expression in B16 and CT26 cells infected with 1 MOI NDV-MIP3α or NDV-WT. (E) Ex vivo chemotaxis for DCs by the supernatants from B16 cells infected with 1 MOI NDV-MIP3α or NDV-WT. (F) In vivo chemotaxis for DCs among the inflammation cells by FCM after the injection of the supernatants (100 µL at 48 hours) from B16 cells infected with 1 MOI NDV-MIP3α or NDV-WT. Data are plotted as mean±SD, *p<0.05, **p<0.01, ***p<0.001. DCs, dendritic cells; FCM, flow cytometry; MIP-3α, macrophage inflammatory protein-3α; MOI, multiplicity of infection; NDV, Newcastle disease virus; NDV-WT, wild-type NDV.

A recombinant NDV expressing the full-length murine MIP-3α (NDV-MIP3α) was engineered in which the murine MIP-3α gene was inserted between the viral P and M genes of the NDV-WT (figure 1A). Several previous results indicated that excessive NDV replication and the resultant robust cell lysis were not necessary for the generation of antitumor immunity. In addition, a prior finding suggested that the expression of a recombinant NDV was the most efficient in the B16 cells.^32^ Thus, we first detected the lytic ability and replicative capacity of NDV-MIP3α in B16 cells. Our data showed that the lytic ability (figure 1B) and replicative capacity (figure 1C) of NDV-MIP3α were equivalent to those of the control NDV-WT in B16 cells. The results presented in figure 1B also show that the moderate titer of NDV-MIP3α for tumor cell lysis was 1 MOI; therefore, to simplify our study, we chose 1 MOI of NDV-MIP3α and its control NDV-WT to perform the subsequent experiments.

The efficient secretion of the MIP-3α protein into the supernatants by B16 and CT26 cells infected with NDV-MIP3α was confirmed by western blot analysis (figure 1D). To detect the biologic activities of the MIP-3α, the supernatants of tumor cells transfected with NDV-MIP3α and NDV-WT were used to perform the DC chemotaxis assay in Transwell chambers. The supernatants from B16 tumor cells transfected with NDV-MIP3α showed a significantly increased chemotactic activity for DCs compared with supernatants of cells infected with the NDV-WT (figure 1E). In addition, the supernatants from both B16 and CT26 tumor cells transfected with NDV-MIP3α were injected into the murine abdominal cavities to observe the chemotactic activity for DCs in vivo. On day 3, after the supernatant injection, all of the inflammatory cells in abdominal cavities were collected and analyzed by FCM. Figure 1F shows the representative images and quantitative analysis results of four experiments, suggesting that there were more CD11c and DEC205 double-positive DCs in the abdominal cavities injected supernatants from NDV-MIP3α-infected tumor cells than in the controls (mice injected with NDV-WT-infected tumor cells, figure 1F). These results indicate that the recombinant NDV-MIP3α virus was successfully constructed, and it effectively secreted bio-active MIP-3α.

### NDV-MIP3α infection induces the production of DAMPs in tumor cells

**Figure 2:**
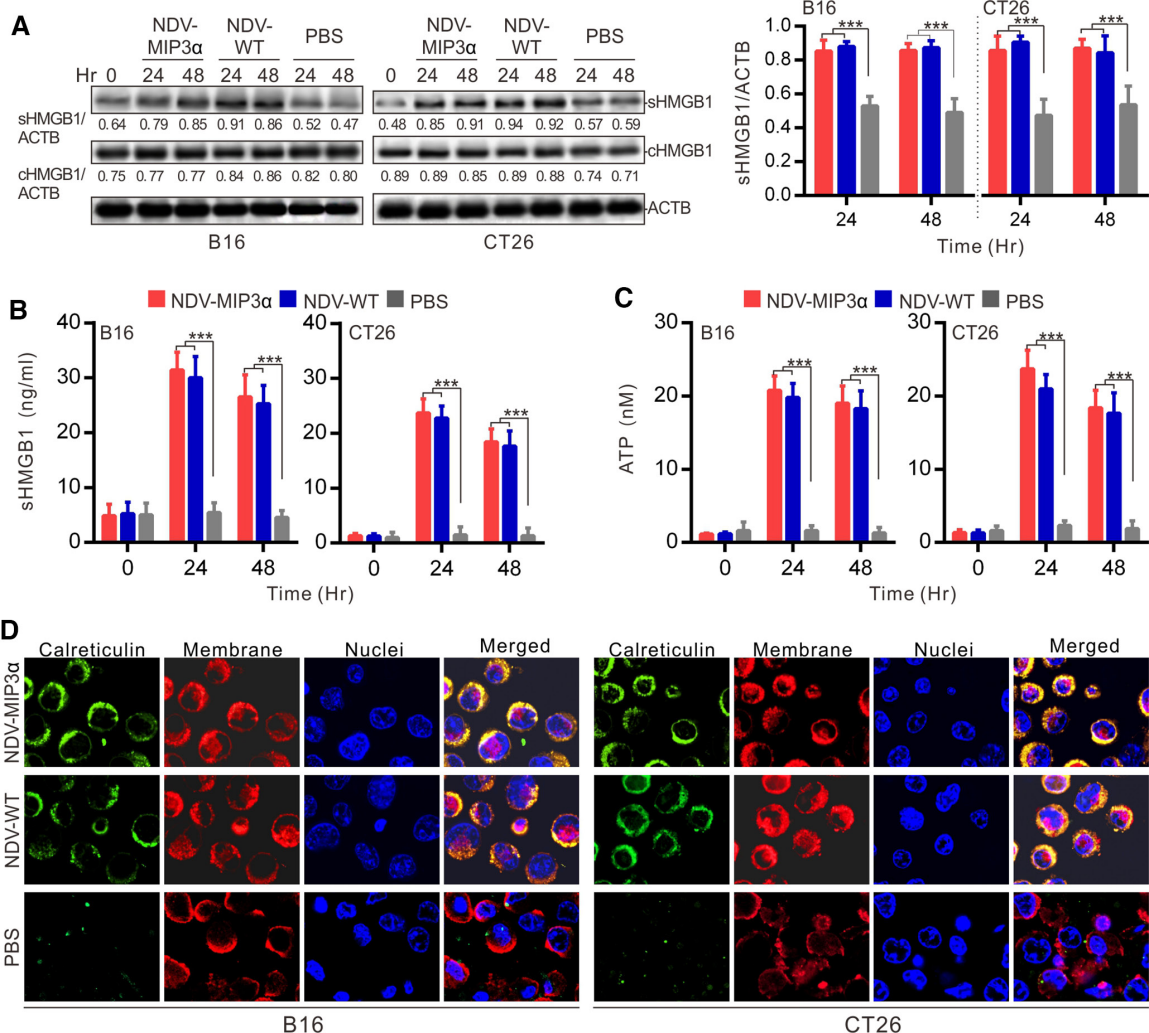
NDV-MIP3α infection induces the production of DAMPs in tumor cells. B16 or CT26 cells were infected with 1 MOI of the indicated NDV or treated with only PBS, and the supernatants were collected at 48 hours after infection. (A) HMGB1 in the supernatant (sHMGB1) and whole-cell (cHMGB1) detected by western blot (left panel) and quantified three experiments with ImageJ software (right panel). (B) The titers of sHMGB1 from three experiments detected by ELISA. (C) ATP released in the supernatants of three experiments quantified by a chemiluminescent determination kit. (D) Ecto-CRT translocation to cell surfaces confirmed by three counterstains and merged analysis with an anti-CRT antibody followed by FITG-conjugated secondary antibody (green), DiI (red), and 4',6-diamidino-2-phenylindole (DAPI, blue). Data are plotted as mean±SD, ***p<0.001. CRT, calreticulin; DAMPs, damage-associated molecular patterns; HMGB1, high mobility group box 1; MIP-3α, macrophage inflammatory protein-3α; MOI, multiplicity of infection; NDV, Newcastle disease virus; NDV-WT, wild-type NDV.

The induction of ICD, which is associated with the DAMP’s production, is considered one of the capabilities of the OV for tumor immunotherapy.^31 36^ Thus, DAMP markers of ICD were detected in B16 and CT26 tumor cells infected with NDV-MIP3α or NDV-WT at 1 MOI. First, the expression and release of the HMGB1 were detected by western blotting and ELISA, respectively. The results showed that the HMGB1 expression (cHMGB1) in the tumor cells (both B16 and CT26) infected with the NDV-MIP3α or NDV-WT and the release to the supernatants (sHMGB1) were almost the same (figure 2A and B). Next, the levels of ATP were detected in the tumor cell supernatants. The results showed that the recombinant NDV-MIP3α and NDV-WT infection significantly increased ATP release in both B16 and CT26 tumor cells compared with the PBS treatment (figure 2C). Finally, the CRT translocated to the outer leaflet of the cell membrane were observed by immunofluorescent staining at 24 hours after the B16 or CT26 cells was infected with NDV-MIP3α or NDV-WT. The results also showed similar levels of CRT translocation onto the cell surface in both B16 and CT26 cells infected with NDV-MIP3α or NDV-WT (figure 2D). Together, these data demonstrate that both NDV-MIP3α and NDV-WT were comparable in their ability to cause DAMP release during the lysis of B16 and CT26 cells, suggesting comparable capabilities of ICD induction.

### NDV-MIP3α infection promotes stronger DC maturation and activation

**Figure 3:**
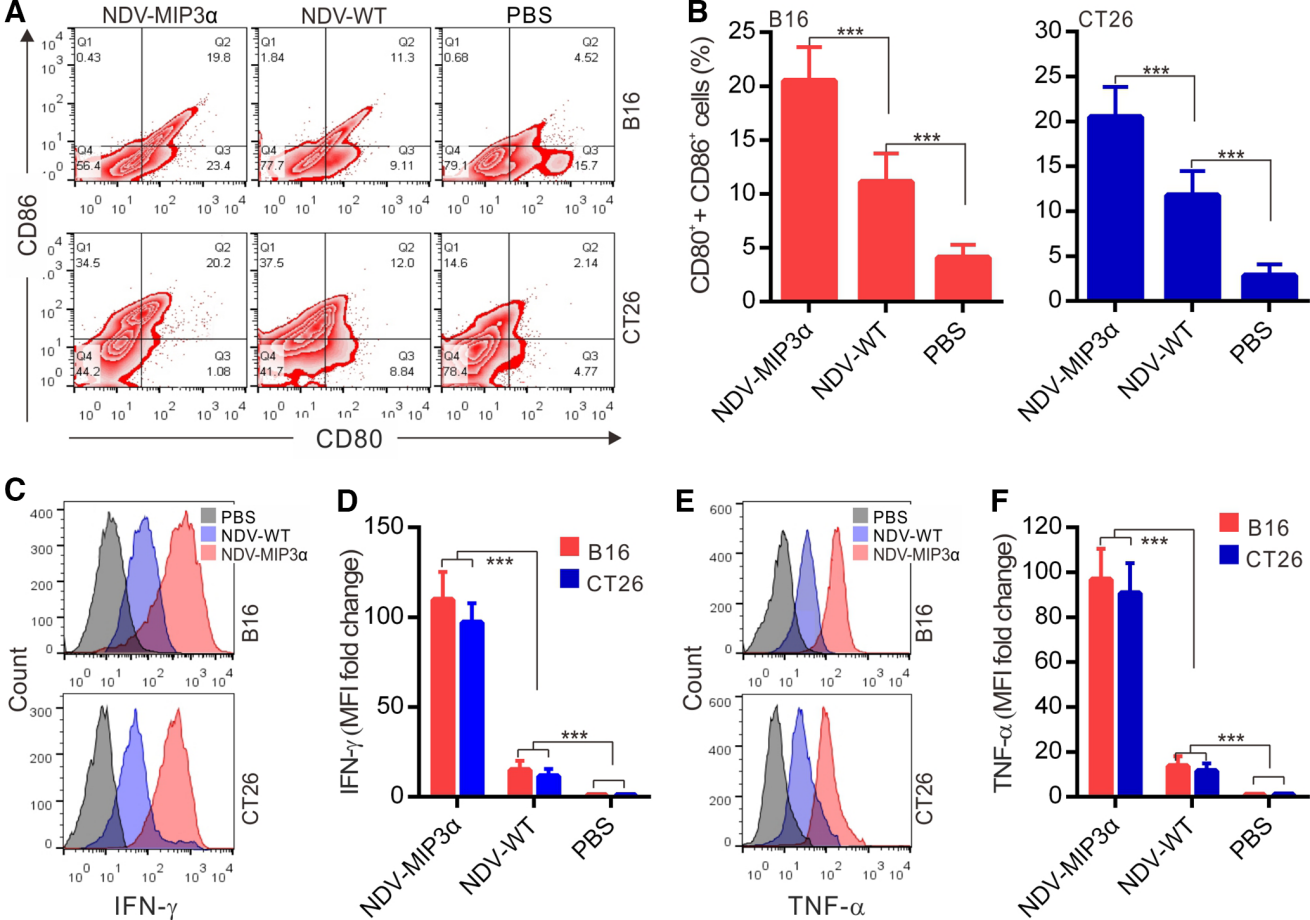
NDV-MIP3α infection promotes DC maturation and activation. mDCs were cultured with the supernatants (50 µL at 48 hours) from B16 or CT26 tumor cells infected with NDV-MIP3α (1 MOI) or NDV-WT (1 MOI), or treated with PBS only for 24 hours. (A) The representative images from FCM analysis of CD80^+^ and CD86^+^ double-positive cells and (B) data of four independent experiments. (C) The representative images of secreting IFN-γ-secreting cells detected by FCM and (D) data of four independent experiments show the MFI fold change normalized to the data of PBS-treated group that is shown as onefold (d). (E) The representative images of secreting TNF-α-secreting cells detected by FCM and (F) data of four independent experiments show the MFI fold change. Data are plotted as mean±SD, ***p<0.001. CRT, calreticulin; DCs, dendritic cells; FCM, flow cytometry; MFI, mean fluorescence intensity. MIP-3α, macrophage inflammatory protein-3α; MOI, multiplicity of infection; NDV, Newcastle disease virus; NDV-WT, wild-type NDV; PBS, phosphate buffer saline.

DC maturation (activation) is the major state of antigen presentation. Thus, DCs isolated from the murine bone marrow were cultured with the supernatants from tumor cells infected with or without NDV for 24 hours. DC maturation markers (CD80 and CD86) and active cytokines (IFN-γ and TNF-α) were detected by FCM. Compared with non-virus-treated (only PBS) DCs, the cell culture supernatants from tumor cells infected with NDV-MIP3α or NDV-WT induced a markedly increased expression of CD80 and CD86, and the most powerful expressions of CD80 and CD86 were found on the DCs treated with the supernatant from the tumor cells infected with NDV-MIP3α (figure 3A and B), indicating the effective maturation of DCs by the supernatant from the tumor cells infected with NDV-MIP3α. Similarly, the supernatants from tumor cells treated with NDV-MIP3α or NDV-WT led to increased secretion of IFN-γ and TNF-α in DCs (figure 3C–F). A more significant secretion of IFN-γ and TNF-α was found in the DCs cocultured with the supernatants from tumor cells infected with NDV-MIP3α compared with the supernatants of cells infected with NDV-WT (figure 3C–F), indicating a great benefit to the activation and differentiation of CD8^+^ and CD4^+^ T cells.

### NDV-MIP3α improves tumor control and enhances DC accumulation

**Figure 4:**
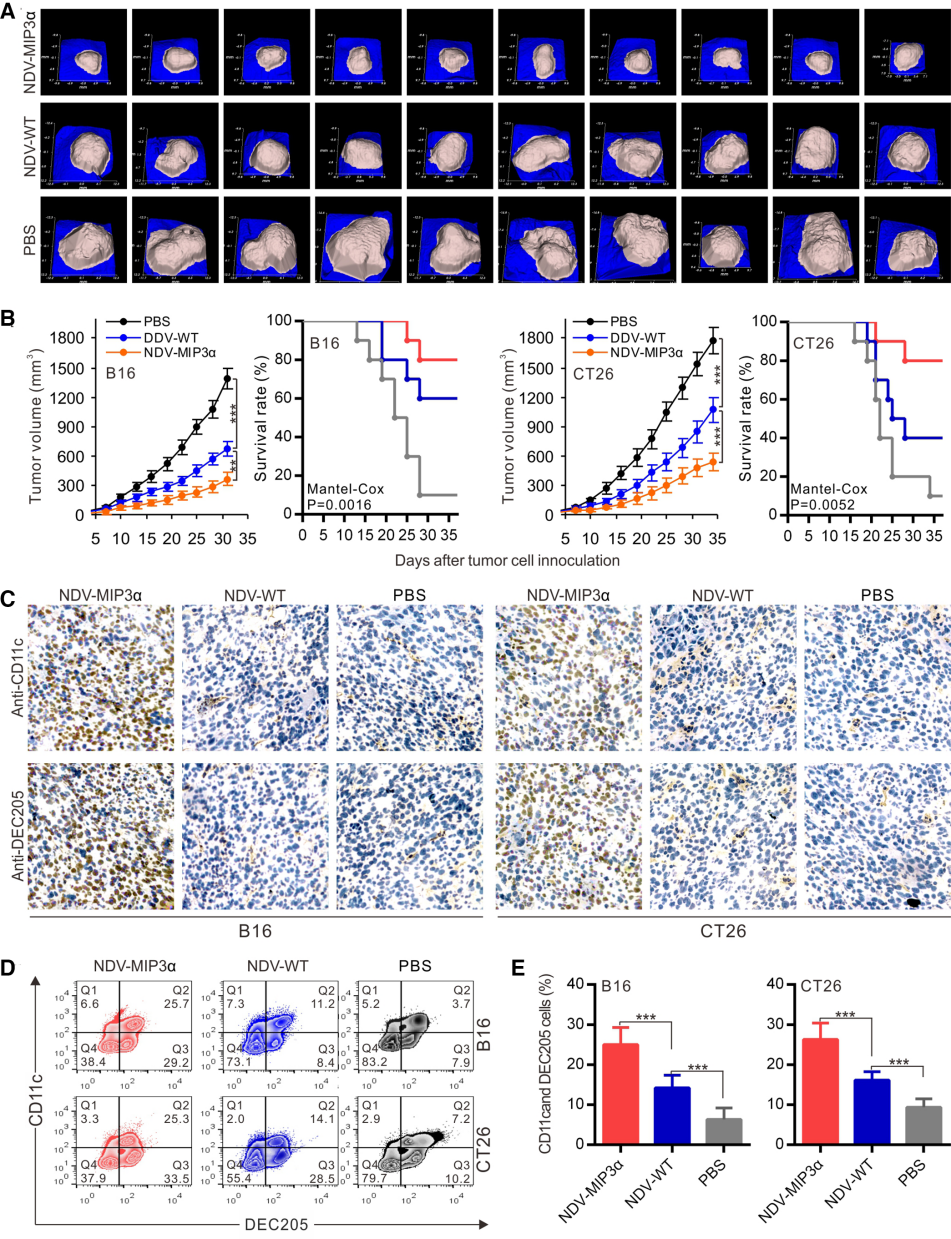
NDV-MIP3α improves tumor control and enhances DC accumulation. B16 or CT26 tumor-bearing mice were intratumorally injected with NDV-MIP3α (2×10^7^ pfu) or NDV-WT (2×10^7^ pfu) or PBS after tumor cell inoculation. The tumor images and volumes were collected by a handheld device (TM900) in a 3-day interval. The tumor tissues were collected on day 25 after tumor cell inoculation. (A) The representative images of B16 tumor masses on day 28 after tumor cell inoculation. (B) Data of the tumor volumes and survival rates of the tumor-bearing mice at the indicated time points in B16 and CT26 tumor-bearing mice (n=10). (C) The representative section images from the indicated tumor masses stained with anti-CD11c and anti-DEC205 antibodies. (D) The representative images of CD11c and DEC205 double-positive TILs detected by FCM and (E) data of three independent experiments. Data are plotted as mean±SD, **p<0.01, ***p<0.001. FCM, flow cytometry; MIP-3α, macrophage inflammatory protein-3α; MOI, multiplicity of infection; NDV, Newcastle disease virus; NDV-WT, wild-type NDV; TILs, tumor-infiltrating lymphocytes.

The therapeutic efficacy of NDV-MIP3α was evaluated in B16 and CT26 tumor models. NDV-MIP3α and control NDV-WT were injected into the B16 tumor masses in C57Bl/6 mice and CT26 in BALB/c mice, when the tumor masses were palpable at about day 5 after tumor cells were injected subcutaneously. Figure 4A presents the images of the tumor masses of B16 mice (n=10) on day 28 after tumor cell injection, showing the smallest tumor masses in mice treated with NDV-MIP3α, compared with the mice treated with NDV-WT or with only PBS. Treatment with NDV-MIP3α and NDV-WT markedly suppressed tumor growth and increased the survival rates of tumor-bearing mice (p<0.001), but treatment with NDV-MIP3α was superior to treatment with NDV-WT (figure 4B). CD11c and DEC205 are two membrane markers of DCs. Thus, to ascertain the accumulation of DCs in tumor tissues, both B16 and CT26 tumor masses at 3 days after the administration of NDV-MIP3α, NDV-WT, or PBS were sectioned and stained with the anti-CD11c and anti-DEC205 antibodies to establish the presence of DCs by immunohistochemistry. Both CD11c and DEC205 positive cells were majorly present in both B16 and CT26 tumor tissues injected with NDV-MIP3α, but not in tumor tissues injected with NDV-WT or PBS (figure 4C). In addition, a more significant infiltration of CD11c and DEC205 double-positive DCs in the tumor tissues of mice treated with NDV-MIP3α was confirmed by FCM analysis (figure 4D and E), suggesting the accumulation of DCs in the B16 and CT26 tumor tissues injected with NDV-MIP3α.

### NDV-MIP3α induces more robust tumor-specific cellular and humoral immune responses

**Figure 5:**
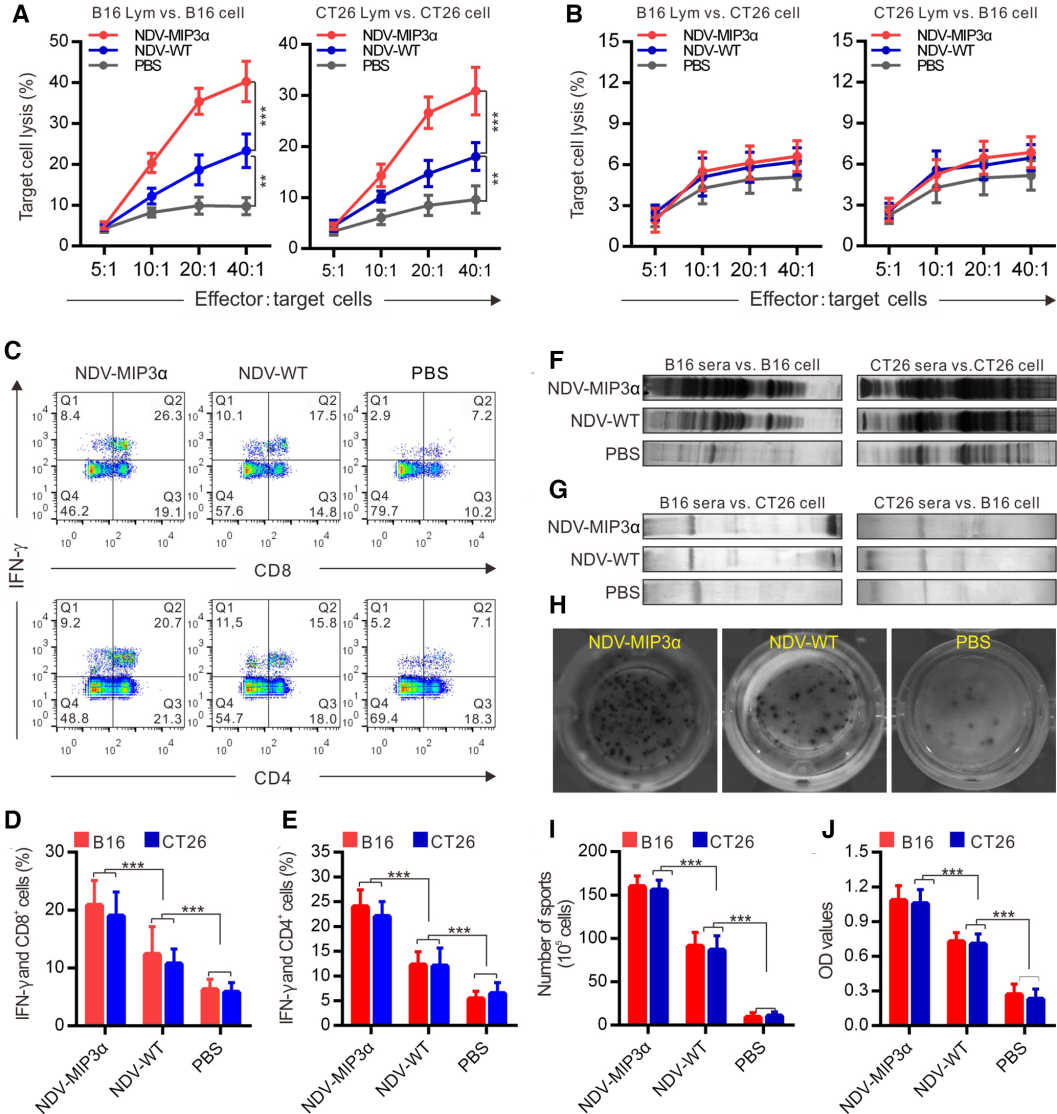
NDV-MIP3α induces stronger tumor-specific cellular and humoral immune responses than the NDV-WT. (A) CTL responses were performed using cells from B16-bearing or CT26-bearing mice treated with the indicated formulations as effector cells, and the B16 or CT26 tumor cells as target cells, respectively. (B) CTL responses using the splenic lymphocytes (Lym) as effector cells, but oppositive tumor cells (B16 Lym vs CT26 and CT26 Lym vs B16) as target cells. (C) The representative images of FCM analyzed IFN-γ-secreted CD4^+^ and CD8^+^ splenic lymphocytes, respectively. (D) The data of four independent experiments of FCM show the percentage of IFN-γ-secreting CD8^+^ lymphocytes. (E) The data of four independent experiments of FCM show the percentage of IFN-γ-secreting CD4^+^ lymphocytes. (F) Western blot using sera IgG from B16-bearing or CT26-bearing mice treated with the indicated formulations against the B16 or CT26 tumor lysates, respectively. (G) Western blot using sera IgG from B16-bearing or CT26-bearing mice against oppositive tumor lysates (B16 sera vs CT26 lysates and CT26 sera vs B16 lysates). (H) The representative images of ELISPOT assay of lymphocytes secreting specific IgG antibodies against B16 cells. (I) The number of lymphocytes secreting IgG antibodies against B16 and CT26 tumor cells detected by ELISPOT assay. (J) The titers of sera IgG specific to B16 and CT26 tumor lysates from mice treated with the indicated formulations detected by ELISA. Data are plotted as mean±SD, **p<0.01, ***p<0.001. CTL, cytotoxic T lymphocyte; ELISPOT, enzyme-linked immunospot; FCM, flow cytometry; MIP-3α, macrophage inflammatory protein-3α; NDV, Newcastle disease virus; NDV-WT, wild-type NDV.

CTLs, especially CD8^+^ lymphocytes, are considered the major component of antitumor immunities induced by tumor vaccines that can perform direct lysis of target tumor cells precisely.^37^ Thus, to evaluate the CTLs specific to B16 and CT26 tumor cells, the splenic monocytes from tumor-bearing mice intratumorally injected with NDV-MIP3α, NDV-WT, or PBS were used as effector cells, and B16 or CT26 cells as target cells. As shown in figure 5A, the splenic monocytes from the B16-bearing mice injected with NDV-MIP3α and NDV-WT showed a markedly increased cytotoxicity to B16 cells, but the splenic monocytes from the NDV-MIP3α-injected mice showed stronger cytotoxicity than NDV-WT-injected mice (figure 5A, left panel). Similarly, the splenic monocytes from the CT26-bearing mice injected with NDV-MIP3α also showed a markedly increased cytotoxicity to CT26 (figure 5A, right panel). To obtain more evidence of tumor-specific CTL activity, the above splenic monocytes from B16-bearing or CT26-bearing mice treated with NDVs were used as effector cells to lyse different tumor cells (B16 lymphocytes vs to CT26 cells, or vice versa). As shown in figure 5B, the splenic monocytes from the B16-bearing mice injected with NDV-MIP3α or NDV-WT did not kill CT26 cells, and the splenic monocytes from the CT26-bearing mice injected with NDV-MIP3α or NDV-WT did not kill B16 cells, suggesting that the CTL activity is tumor-specific. In addition, splenic monocytes from mice injected with NDV-MIP3α, NDV-WT, or PBS were doubly stained against IFN-γ and CD8 (or CD4) and then analyzed by FCM. The percentages of both CD8^+^ and CD4^+^ T cells expressing IFN-γ were markedly increased in the mice injected with NDV-MIP3α and NDV-WT, with the highest percentages in NDV-MIP3α-injected mice; the number of CD8^+^ and CD4^+^ T cells expressing IFN-γ in the NDV-MIP3α-injected mice increased about onefold to 1.5-fold compared with PBS-injected mice and about onefold compared with NDV-WT-injected mice (figure 5C–E).

To investigate whether NDV-MIP3α enhanced the production of antibodies, we first detected the IgG antibodies in sera from mice injected with NDV-MIP3α, NDV-WT, or PBS by western blotting analysis. We found that all of the sera from mice injected with NDV-MIP3α, NDV-WT, or PBS contained different levels of antibodies against B16 and CT26 tumor cells (figure 5F). However, compared with the sera from mice injected with PBS, although an increased level of antibody response was detected in the sera from mice injected with NDV-WT, a much stronger antibody response was found in the sera from mice injected with NDV-MIP3α (figure 5F). To observe whether the antibody response was tumor-specific, the sera from the B16-bearing or CT26-bearing mice treated with NDVs were used to detect different tumor lysates. Our data showed that the sera from B16-bearing mice did not recognize CT26 cell lysate, and conversely, CT26 sera did not recognize B16 lysate (figure 5G), suggesting the antibody response was also tumor-specific. In addition, we measured the numbers of splenic monocytes secreting specific IgG antibodies against B16 or CT26 cells by ELISPOT assay. Although the number of splenic monocytes secreting specific IgG antibodies against B16 or CT26 cells increased in the mice injected with NDV-MIP3α and NDV-WT compared with PBS-injected mice, but the number of splenic IgG-secreting monocytes in the NDV-MIP3α-injected mice increased more significantly (p<0.001), about onefold increase than that in the NDV-WT-injected mice (figure 5h and i). Moreover, a considerably increased antibody response was found in the sera from mice injected with NDV-MIP3α by ELISA detection, about a onefold increase compared with the NDV-WT-injected mice and a threefold increase compared with the PBS-injected mice were observed (figure 5J). Taken together, these data indicate that injection of NDV-MIP3α induces the production of tumor cell-specific cellular and humoral immunities.

### Adoptive transfer of splenic lymphocytes or antibodies suppresses tumor growth

**Figure 6:**
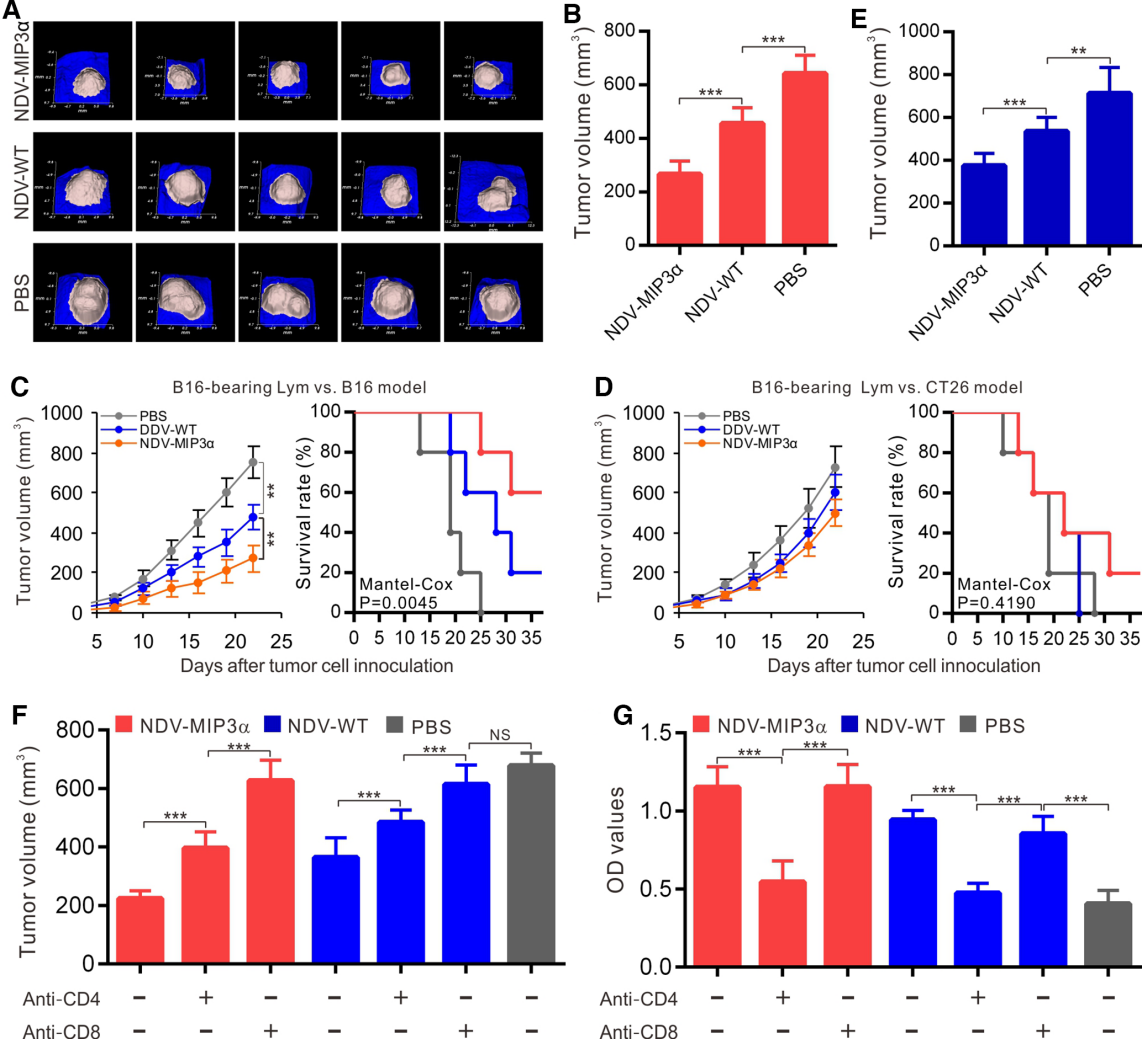
Antitumor activities by adoptive transfer of splenocytes and antibodies, and the role of T cells. (A and B) Adoptive transfer of splenocytes from B16-bearing mice treated with the indicated formulations to the recipient syngeneic B16-bearing mice (n=5). Data show the tumor images (A) and tumor volume (B) on day 18 after tumor cell challenge. (C) Adoptive transfer of splenocytes from the mice treated with indicated formulations to the recipient syngeneic B16-bearing mice (n=5). Data show the tumor volumes and survival rates at the indicated time points. (D) Adoptive transfer of splenocytes from the B16-bearing mice treated with indicated formulations to the recipient heterogeneic CT26-bearing mice (n=5). Data show the tumor volumes and survival rates at the indicated time points. (E) Adoptive transfer of antibodies (50 mg/kg) from mice treated with indicated formulations to recipient syngeneic B16-bearing mice (n=5). Data showed the tumor volumes on day 18. (F) The CD4 or CD8 T-cell subsets depleted by intraperitoneal injection of mAb (50 mg/kg per mouse) into B16-bearing mice treated with the indicated NDV or PBS. The data show the tumor volume on day 18 after tumor cell injection. (G) The CD4 or CD8 T-cell subsets depleted by injection of mAb in B16-bearing mice treated with the indicated NDV or PBS; the data show the titers of IgG antibodies in sera of B16-bearing mice on day 18 after tumor cell inoculation by ELISA. Data are plotted as mean±SD, **p<0.01, ***p<0.001. mAb, monoclonal antibodies; MIP-3α, macrophage inflammatory protein-3α; NDV, Newcastle disease virus; NDV-WT, wild-type NDV.

To get direct evidence that the antitumor activities induced by NDV-MIP3α are related to the cellular and humoral immune responses, we isolated splenic lymphocytes and antibodies from mice injected with NDV-MIP3α, NDV-WT, or PBS. We adoptively transferred them into newly established B16-bearing mice to monitor the tumor growth. To observe the specific CTL response, tumor cells were first inoculated into mice, and splenic monocytes were intraperitoneally transferred on day 3 for three times in 3-day intervals. The tumor images (figure 6A) and volumes (figure 6B) were captured by Peria TM900 on day 18 after tumor cell inoculation. The results indicated a more significant decrease in tumor volume in the mice injected with the monocytes from the NDV-MIP3α-injected mice (figure 6B), compared with the NDV-WT-injected or PBS-treated mice. In addition, our results showed that the transfer of lymphocytes from B16-bearing mice treated with NDV-MIP3α significantly inhibited the long-term tumor growth and increased survival of B16-bearing mice (figure 6C). However, the transfer of lymphocytes from B16-bearing mice treated with NDV-MIP3α had no effect on CT26-bearing mice (figure 6D), suggesting that the CTL activities are tumor-specific. To observe the antitumor effects of the antibodies induced by different NDVs, antibodies (50 mg/kg) from the mice injected with NDV-MIP3α, NDV-WT, or PBS were injected intraperitoneally into the B16 model mice after the tumor masses were palpable (about 3 days after cell inoculation). Antibody injection was given three times in 3-day intervals, and the tumor volumes were evaluated on day 15 after the adoptive transfer of antibodies. Our results showed that the transfer of antibodies from the NDV-MIP3α-injected or NDV-WT-injected mice significantly suppressed the tumor growth compared with the PBS-treated mice (p<0.001), but this phenomenon was more significant in the mice injected with antibodies from the NDV-MIP3α-injected mice (figure 6E).

### CD8^+^ and CD4^+^ T cells play important roles in the induction of antitumor immunity

The roles of the CD4^+^ and CD8^+^ T cells in the production of antitumor immunities induced by NDV-MIP3α were explored in this study. The depletion of CD4^+^ and CD8^+^ T cells was performed by intraperitoneal injection of anti-CD4^+^ or anti-CD8^+^ monoclonal antibodies, respectively, into B16-bearing mice. Tumor volumes were then observed, and the titer of the tumor-specific anti-IgG was detected by ELISA. Compared with the PBS-treated mice, significant tumor growth was found in the mice depleted of CD4^+^ or CD8^+^ cells. Still, more significant tumor growth was found in the latter, suggesting that CD8^+^ T cells take the most important role in the production of antitumor immunity induced by NDV-MIP3α and that CD4^+^ cells are partially related to the production of antitumor immunity (figure 6F). In addition, the level of the antibody subset specific to B16 cells in the sera from mice depleted of CD4^+^ T cells was significantly decreased almost to the level of the mice injected with PBS, but this was not related to the CD8^+^ T cells (figure 6G). These results indicate that both CD4^+^ and CD8^+^ T cells are involved in the production of antitumor immunity induced by NDV-MIP3α, but CD8^+^ T cells play a more critical role in the process.

### NDV-MIP3α modulates the tumor microenvironments

**Figure 7:**
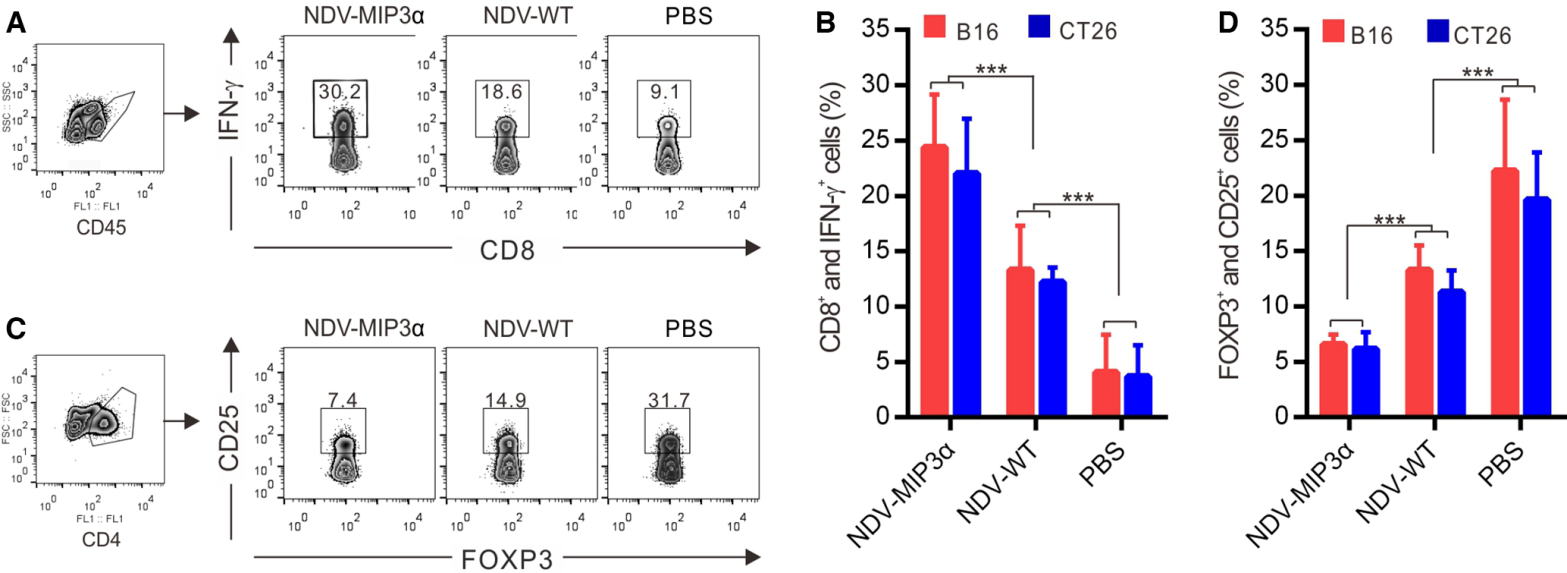
NDV-MIP3α modulates the tumor microenvironments. The TILs were isolated from the tumor masses of B16-bearing and CT26-bearing mice, CD45 or CD4 cells were gated as indicated and analyzed by FCM. (A) The representative images of FCM-analyzed CD45^+^ and CD8^+^ lymphocytes secreting IFN-γ in the tumor-bearing mice treated with the indicated NDV or PBS. (B) The data of four independent experiments of flow cytometry (FCM) show the percentage of CD45^+^ and CD8^+^ lymphocytes secreting IFN-γ. (C) Representative images of FCM-analyzed CD4^+^, CD25^+^, and Foxp3^+^ triple-positive Tregs in the mice treated with the indicated NDV or PBS. (D) The data of four independent experiments of FCM show the percentage of CD4^+^, CD25^+^, and Foxp3^+^ triple-positive Tregs. Data are plotted as mean±SD, ***p<0.001. FCM, flow cytometry; MIP-3α, macrophage inflammatory protein-3α; NDV, Newcastle disease virus; NDV-WT, wild-type NDV; TILs, tumor-infiltrating lymphocytes; Tregs, regulatory T cells.

An immunosuppressive microenvironment is one of the strategies that tumors develop to escape immune surveillance, leading to uncontrolled malignant growth.^38^ Thus, the phenotypes of the TILs in the tumor tissues of both B16 and CT26 models were detected by FCM. The active IFN-γ-secreting CD8^+^ T lymphocytes were found to be in significantly higher numbers in the tumor tissues from the NDV-MIP3α-injected mice than in the tissues from NDV-WT-injected mice (onefold difference) and the PBS-treated mice (sevenfold, figure 7A and B). Conversely, the number of suppressive CD25^+^ and FOXP3^+^ double-positive Tregs significantly decreased in the tumor tissues from the NDV-MIP3α-injected mice, about onefold compared with the NDV-WT-injected mice and about threefold compared with the PBS-injected mice (figure 7C and D). These results suggest that NDV-MIP3α injection reverses the immunosuppressive microenvironment, as evidenced by the decrease in suppressive Tregs and the increase in active IFN-γ-secreting CD8^+^ cells.

## Discussion

Infection and replication of OVs in tumor tissues can lead to the direct lysis of tumor cells, production of IFN-α, reversion of the suppressive tumor microenvironment, and induction of ICD against tumor cells through the release of DAMP molecules and tumor-associated antigens.^39^ Gene-engineered OVs expressing immunomodulatory molecules, such as MIP-3α in this study, offer an additional opportunity to increase the intratumoral delivery and spread danger-associated and damage-associated pattern signals to the innate immune effectors. Recombinant OVs engineered with a GM-CSF gene are currently the most advanced tools in clinical development to enhance innate immune responses, which have been recently approved by the USA Food and Drug Administration for the treatment of metastatic melanoma.^39^ Despite many signs of progress, the appropriate immunomodulatory targets for intratumoral OV-based therapy are still unknown. In addition, OV-based therapy is influenced by the replicative capacity, individual OV biology, and the interaction of the OVs with the tumor and stromal cells.^40^ Thus, in this study, we further hypothesized that intratumor expression of the MIP-3α gene using a recombinant oncolytic NDV would result in the attraction of peripheral DCs into the virus-infected tumors and subsequently lead to the priming of a stronger and more specific cellular and humoral immune response against tumors that will inhibit tumor growth and promote the survival of the host. The results of this study validate our hypothesis. In B16 and CT26 tumor models, intratumor injection of the NDV-MIP3α-induced specific cellular and humoral immunities that significantly suppressed the tumor growth. The recombinant (NDV-MIP3α) and wild-type (NDV-WT) viruses had almost the same abilities to lyse tumor cells and induce ICD ex vivo. NDV-MIP3α expressing MIP-3α was shown to have the capability of attracting DCs *ex* and in vivo. NDV-MIP3α-infected tumor cells promoted a strong DC activation and maturation. The administration of NDV-MIP3α markedly attracted DCs to the tumor tissues, increased the number of IFN-γ-secreting CD8^+^ lymphocytes in the tumor tissues, and sufficiently primed specific CTLs to protect naive mice from a subsequent tumor challenge by adoptive transfer of splenocytes from NDV-MIP3α-injected mice. In CD8-depleted mice, tumor growth in the NDV-MIP3α-treated mice recovered to almost the same as in the PBS-treated mice, suggesting the CD8-dependent immunity plays a vital role in the antitumor effect.

DCs, which are the most powerful antigen-presenting cells, can be found almost anywhere in the body, but especially where the foreign pathogens easily invade.^41^ DCs are the major activators of T cells and initiate specific adapted immune responses after exposure to antigens. DCs endocytose the antigens and subsequently process and present the antigen epitopes onto the cell membranes, and then migrate to the T-cell areas of lymphoid tissues.^41 42^ After entering the lymphoid tissues, DCs mature by undergoing a series of changes in phenotype and function.^41 42^ Immature DCs capture and process soluble-protein antigens more efficiently, but mature DCs are more capable to prime naive T cells.^41 42^ Up to now, a variety of mediators have been found to have the ability to regulate DC migration, including PAMPs, TNF-α, IL-1β, and GM-CSF. However, the relative importance and the detailed molecular mechanisms of these mediators in vivo are still not fully understood.^43–45^ Recent research progress indicates that some chemokines, including the MIPs (MIP-1α, MIP-1β, and MIP-3α), monocyte chemotactic proteins (MCP-1, MCP-2, MCP-3, MCP-4, and MIP-5), and CC chemokines RANTES, are very important for the directional migration of DCs to secondary lymphoid tissues.^45–48^ MIP-3α is a CC chemokine that can attract DCs and lymphocytes but cannot attract monocytes or neutrophils.^48^ Immature DCs originate from CD34-positive hematopoietic progenitor cells sensitively, specifically respond to MIP-3α, and subsequently migrate vigorously to lymphatic tissues. However, mature DCs lose their abilities to respond to MIP-3α.^48^ MIP-3α is majorly expressed in the lung, liver, and lymphoid tissues, as well as in endothelial cells and monocytes after these tissues are invaded by pathogens, or the cells are exposed to inflammatory stimuli such as LPS or TNF-α.^48 49^ In our current study, we found that there was no MIP-3α expression in both the B16 and CT26 tumor cells ex vivo and tumor tissues of tumor-bearing mice in vivo. However, after the infection of NDV-MIP3α ex vivo or the injection of NDV-MIP3α in vivo, a significant expression of NDV-MIP3α was found in the tumor cells and tumor tissues. Moreover, MIP-3α expressed by NDV-MIP3α both ex vivo and in vivo could still attract DCs, a finding consistent with a previous study,^49^ suggesting that the MIP-3α expressed by the recombinant NDV-MIP3α plays an important role in promoting the antitumor activities reported here.

DCs and other immune cells recognize and quickly respond to pathogen-infected cells through PAMPs. PAMPs exposed by infected cells can be recognized by immune cells and result in the immune cell-mediated killing, phagocytosis, and subsequent antigen presentation by DCs or other antigen-presenting cells.^12–14^ Viral and bacterial components, including certain nucleic-acid sequences (eg, CpG), are the most classic examples of PAMPs. In addition to the response to pathogens, appropriate immune responses occur in the abnormally dying or stressing cells (eg, malignant precancerous cells). In 1994, Polly Matzinger proposed the revolutionary ‘danger theory,’ which greatly promoted the subsequent establishment of ICD theory.^50^ The theory states that the immune system distinguishes not only danger signals induced by non-self-pathogens or other substances but also the self-endogenous danger signals. ICD of cancer cells can expose DAMPs as self-endogenous danger signals for the host immune system.^8–11^ Both PAMPs and DAMPs are ligands of pattern recognition receptors (PRRs), such as Toll-like receptors, which are inherently present in immune cells.^8–11^ PRR recognition of PAMP or DAMP ligands leads to downstream effects such as the differentiation of immune cells and the production of cytokines, which ignite immune responses localized around abnormal tissues. Because oncolytic NDV inherently can direct the lysis and ICD induction of tumor cells, we thus hypothesized that if NDV was additionally able to produce the DC chemokine MIP-3α by recombinant gene-engineering, then the intratumoral injection of the recombinant NDV (NDV-MIP3α) could directly produce MIP-3α in the tumor tissues. This effect would result in the accumulation of DCs within the tumor tissues and the in vivo interaction of DCs with the PAMP/DAMP molecules and tumor lysates/tumor antigens. We further hypothesized that the recombinant NDV-MIP3α should induce more powerful tumor-specific immunities against the tumor with a consequently stronger suppression of tumor growth. In this study, we demonstrated that local production of MIP-3α by NDV-MIP3α injection in the tumor tissues could induce the intratumoral accumulation of DCs with a stronger tumor-specific CTL and antibody response and more significant suppression of tumor growth without ex vivo manipulation of DCs. Interestingly, this strategy also induced the modification of the tumor microenvironment that was evidenced by the increased number of INF-γ-secreted CD8^+^ lymphocytes and the decreased number of Treg lymphocytes. Therefore, the strategy for the generation of in vivo DC vaccines in the current study may help boost the antitumor immunity for the treatment of cancer.

## Conclusions

The findings in this study prove the principle that immunotherapy with an oncolytic NDV expressing the DC chemokine MIP-3α (NDV-MIP3α) enhances the production of antitumor immunities and reversely modulates the tumor immunosuppressive microenvironments. The recombinant NDV-MIP3α keeps the capacities of NDV-WT on tumor lysis and ICD induction. The stronger antitumor immunities induced by NDV-MIP3α compared with the NDV-WT are related to the increased promotion of DC maturation and infiltration into tumor tissues. The CD8^+^ subset plays a vital role in the production of the specific antitumor immunities. The findings offer a strong rationale for further clinical evaluation of in vivo DC vaccine strategies, such as the intratumoral injection of the recombinant NDV-MIP3α described here.
